# Conisation course for medical students-experience from a German University Hospital

**DOI:** 10.4274/jtgga.galenos.2019.2019.0126

**Published:** 2020-06-08

**Authors:** Ferenc Zoltan Takacs, Erich-Franz Solomayer, Amr Hamza, Ingolf Juhasz-Böss, Panagiotis Sklavounos, Julia Caroline Radosa, Sebastian Findeklee

**Affiliations:** 1 Department of Gynecology, Obstetrics and Reproductive Medicine, Saarland University Hospital, Homburg, Germany

**Keywords:** Conisation simulator, student teaching, questionnaire

## Abstract

**Objective:**

Conisation of the cervix is one of the most common surgical procedures in gynaecology. Nevertheless, surgical expertise is required because if the cone is too small, the oncological risk increases and if the cone is too large, the obstetric risk increases. The aim of this prospective study was to investigate the suitability of an in-house conisation simulator for teaching medical students the practical performance of conisation.

**Material and Methods:**

Following a demonstration, students performed a loop conisation with a target depth of 8-10 mm using the simulator. Cone biopsy dimensions were analysed and a loop electrosurgical excision procedure (LEEP) score was calculated. The students were surveyed using a questionnaire of 12 items with five possible responses for each in order to investigate the suitability and realism of the teaching experience.

**Results:**

Eighty-nine students participated in the course. The median (range) cone depth was 8 (3-25) mm with a standard deviation of 3.3 mm. The observed LEEP score amounted to 1.5. The questionnaire was answered by 88 students and completed by 86. Survey results showed the course was consistently rated as positive, especially towards the increase in practical skills. The questionnaire item producing the highest score was “I enjoyed the course” while the statement “I have gained enough self-confidence for the application of high-frequency surgery” received the lowest approval score. Students considered the course to be realistic and a helpful teaching exercise.

**Conclusion:**

Practical surgery exercises on the surgical simulator were received positively. Simulation training could be extended to other gynaecological operations and to other medical subjects.

## Introduction

Conisation is one of the minor gynaecological operations and in addition, it is one of the most common surgical procedures in gynaecology. Therefore, it is considered a typical operation for beginners but the procedure cannot be regarded as trivial. The removal of a too small a cone when excising diseased tissue may be at the expense oncological safety, thus requiring follow-up operations and/or therapies (1). Conversely, removing too large a cone will increase the oncological safety but will also increase the patient's obstetric risk in the event of a subsequent pregnancy. It has been reported that the risk of cervical insufficiency and consecutive premature birth in pregnancy after conisation is about 25% (2,3). This is aggravated by the fact that cervical dysplasia in need of treatment mostly occurs in young women aged 30-35 years (4).

How should one deal with this dilemma in clinical practice? Preventing young colleagues from performing conisations is not possible - because eventually there would not be the specialists capable of conisation. However, patient safety is paramount.

Therefore, a simulator for practicing conisation with an electric loop was developed at our center (5). This was then used during gynaecological and obstetric practical clerkship training. Students were asked to attempt an optimal conisation, following a demonstration by the doctor in charge of the study (study doctor). The students were then surveyed concerning the experience of practical training.

## Material and Methods

The conisation simulator was used as part of the gynaecology and obstetrics practical clerkship at our gynaecology clinic in the summer semester 2018 (examination period: 13.04.2018-13.07.2018). This was a prospective study with fifth year medical students. The only inclusion criterion was participation in the conisation simulation course. The students were informed in detail about the study before participating. Participation was voluntary and anonymous. The study was previously approved by the Local Ethics Committee (approval number: 259/17).

### Conisation simulator

**Figure 1:**
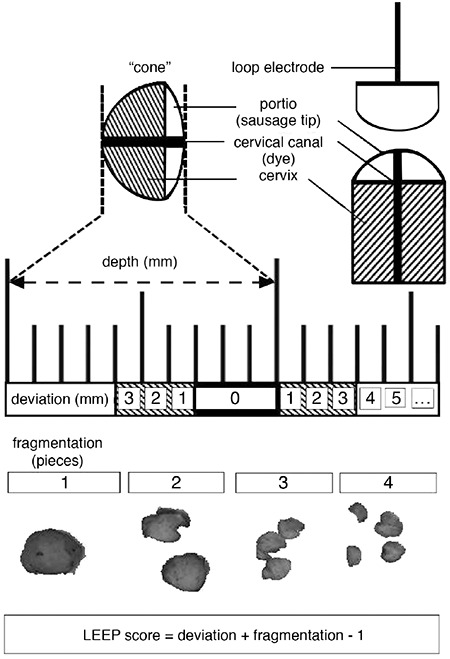
Scheme of the loop electrosurgical excision procedure simulator LEEP: Loop electrosurgical excision procedure

**Figure 2:**
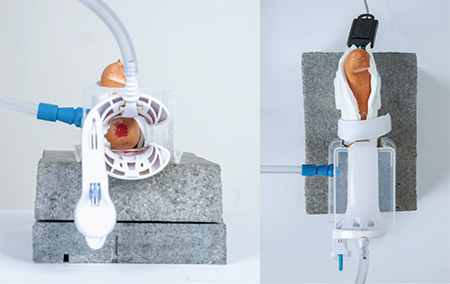
Construction of the conisation simulator

A loop electrosurgical excision procedure (LEEP) was performed on the simulator. The conisation simulator was a table-top model with a self-holding speculum. A stone slab formed a stable surface, and a polystyrene plate lying on top of it conformed well to the shape of the speculum. A self-holding speculum with smoke evacuation was ideal for performing a LEEP under local anaesthesia and realistic conditions. For the simulation of the portio the end of a sausage was used. The cervical canal was visualized by the injection of red dye. Thus, the fragmentation and thickness of the cone could be better illustrated. The sausage was placed directly on the neutral electrode and fixed with a Velcro bandage (see Figure 1, 2). This allowed numerous quick repetitions. The LEEP was performed with the monopolar power device ERBE Vio300D (Erbe Elektromedizin GmbH, Tübingen, Germany) with a loop electrode (Erbe Elektromedizin GmbH, Tübingen, Germany) under colposcopic view (Olympus OCS-500, Olympus Europe, Hamburg, Germany) as previously described (5).

The course took place on the last day of the one-week practical clerkship and lasted 30 minutes. First, the study doctor carried out a loop conisation. Subsequently, the students in groups of around eight at a time, had the opportunity to make a loop conisation and up to two post-resections on their own under supervision by the study doctor.

After the course, the study doctor, who was a specialist in obstetrics and gynaecology employed at the study center, was asked if the simulator was suitable for everyday use and the course as realistic and if he could imagine assisting the students in a LEEP.

### Loop electrosurgical excision procedure score for excision

single cut. The specimens were measured with a digital calliper in the area of the cervical canal and visualized with dye. Depending on the depth and shape of the specimen, the students were able to perform a subsequent resection. The thickness of each specimen was added to obtain the total cone thickness. Thus even if each individual cut showed a large deviation from the target range, resections could still result in a normal mean thickness. To record the excisions that missed the target range of 8-10 mm, deviations were recorded separately. The deviation from the desired cutting depth was calculated as follows. If the specimen thickness was between 8 and 10 mm, the deviation was 0. For superficial cuts, that is those less than 8 mm, deviation from the desired minimum was recorded so that a 6 mm specimen would have a depth deviation of 2 mm. Similarly for a cut that was too deep, that is greater than 10 mm, deviation from the desired maximum was recorded so that an 11 mm specimen would have a deviation of 1 mm. In order to account for both fragmentation and deviation together, a LEEP score was calculated as recently described (5).

### Statistical analysis

All variables were analysed descriptively using median and standard deviation (SD) for continuous variables. Data was analysed using an electronic database (Microsoft Office Professional, Excel version 2007, Redmond, Washington, USA).

### Study questionnaire

Students were asked to complete a self-developed anonymous questionnaire for the evaluation of the event, directly after performing the conisation. Participants were asked to rate the following statements using five possible grades (“agree”, “agree somewhat”, “neutral”, “disagree somewhat” and “disagree”). The following 12 statements used to evaluate the course:

1. The course has improved my operational skills.

2. The course helps me in dealing with patients.

3. The course has improved my medical study quality.

4. I wish to do more operation simulation exercises in the practical year.

5. I wish to perform more operation simulation exercises in other subjects.

6. The surgical simulation improves my understanding of the subject gynaecology and obstetrics.

7. The course improves my competence in gynaecology and obstetrics.

8. The surgical simulation has expanded my competence in gynaecological examination.

9. I have received sufficient knowledge about high-frequency surgery.

10. I have enough confidence to perform high-frequency surgery myself due to the course.

11. I could carry out a LEEP under supervision myself.

12. I enjoyed the course.

## Results

A total of 89 out of 90 medical students performed a conisation with the simulator. One person could not attend for health reasons.

The median (range) total cone depth during the 89 conisations was 8 (3-25) mm and the SD was ±3.3 mm with 34 (38.2%) conisations being too superficial and 14 (15.7%) too deep. Thus, 41/89 students (46.1%) achieved the target range for cone depth of 8-10 mm with one conisation and 64/89 students (71.9%) reached the target range with additional subsequent resection. A total of 34 subsequent resections with a median (range) depth of 5 (2-10) mm and a SD of ±1.9 mm were performed. 25/89 (28.1%) did not reach the target range. We observed a LEEP score of 1.5 for the 89 medical students.

Out of the 89 students, 88 completed questionnaire and 86 forms were completely filled. On two questionnaires one answer was missing.

The study doctor assessed the conisation simulation course in all 89 cases as suitable for everyday use as part of normal student teaching. Furthermore, the course was perceived as realistic and the study doctor was confident in assisting a loop conisation in the operation room for all 89 medical students after the course.

The students’ conclusions regarding the teaching experience were consistently positive. Table 1 summarizes the results of the course evaluation by the medical students. The highest rated aspect of the course was enjoyment of the course with nearly 91% complete approval. The item with the worst assessment by the students concerned having enough self-confidence for performance of high frequency surgery on their own, with only 30% complete approval.

## Discussion

To our knowledge, this is the first study ever examining a conisation simulator in the context of student teaching. The conisation exercises with the Homburger conisation simulator were rated almost entirely positive by both the study doctor and the participating students. An indication of this finding can be seen in the fact that for all questions, the first answer category (“agree”) was most often chosen although this was equal with the neutral response to the statement “self-confidence in the application of high frequency surgery”. However, it also seems interesting that the students’ answers were by no means homogeneous. This increases the validity of the answers, since evaluations within the framework of student teaching run the risk that the same answer will always be chosen or overestimated because of a lack of interest, in order not to disappoint the teachers.

Three basic tendencies can be seen in the analysis. First of all, the practical operation simulation exercise was very well received by the students, were perceived as a lot of fun and there was a strong desire to implement more simulation exercises during their studies. Second, they seem to bring about a global increase in knowledge, both in the theoretical and practical fields, with the practical gain in knowledge appearing to be greater than the theoretical one. Third, the students still expressed reservations about the practical application of surgical techniques to the patient under everyday clinical conditions as was evident by the lowest positive response (35.2%) concerning the self-confidence gained in using the methods of high frequency surgery in real practice. It must be emphasized that this can hardly be expected from a 30 minute course. It should also be kept in mind that, from experience, only half of the students will be interested in working in an operative subject later. Among future gynaecologists, the approval rates might have been higher.

The fact that the majority of students were able to reach the target range for conisation, with the aid of a subsequent resection if required, can be regarded as an encouraging result. However, the conisation simulator should be evaluated in further studies, in particular the impact of repetitive training on conisation depth, LEEP score and surgeon’s self-confidence should be investigated further.

In this study practical exercises as an element of medical student teaching were investigated. These practical exercises can take several forms. They can be performed on humans (usually patients) as well as on animals, for example the practice of complex surgeries or interventional procedures such as heart valve replacement, as well as on models specially created for an intervention, as in this study (6,7,8). Undoubtedly, a non-living model is the most favourable solution, because it minimises ethical concerns. Additionally, most models would have unlimited reusability. An open question is the financing of simulation training. Unfortunately, not all university hospitals have a sufficient teaching budget to provide such models in sufficient numbers. One reason for this could be that practical exercises, especially of surgical interventions, are not yet an integral part of the curriculum within the clinical section of medical studies. If this were the case, medical schools would have a greater incentive to provide funding for it.

Alternative teaching concepts for practical exercises using simulation models also provide theoretical knowledge transfer, for example in the context of a lecture or a seminar and showing techniques with the help of various media, such as pictures or videos (9,10). In this case a practical simulation exercise was deliberately chosen because we believe that surgical procedures are best learned by actually experiencing the procedure and by repeating the procedures in a work or simulation setting. The publication by Spüntrup et al. (11) which showed that endoscopic surgery can be learned through repeated practice, confirmed the feasibility of the concept. In addition, we believe it is unethical to practice operations primarily on humans or animals.

## Conclusion

It is suggested that the conisation simulator for learning LEEP by medical students as well as physicians in further education has merit and further study is warranted.

We conclude that surgical simulation exercises, including exercises for the implementation of loop conisations, can be carried out without problems under everyday conditions in a university hospital and are rated positively by both the teacher and the students. With the aid of simulators practical surgical skills as well as theoretical knowledge can be taught efficiently. We propose that operation simulation exercises should be used much more widely, not only in gynaecology but also in other subjects, and that it may be possible to extend them to other operations or scientific issues.

## Figures and Tables

**Table 1:**
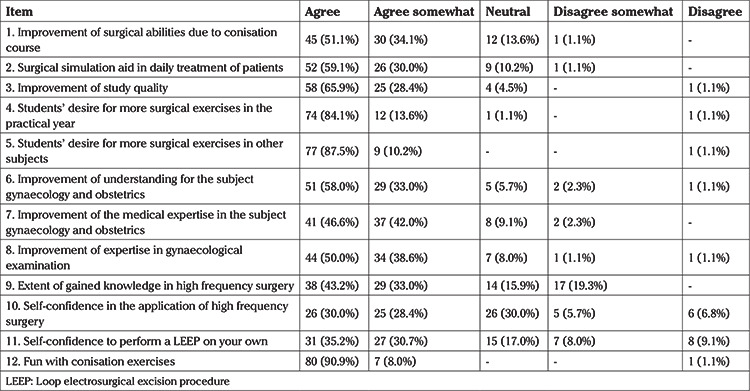
Evaluation of the conisation course by the medical students (n=88)
